# Punicic acid: A striking health substance to combat metabolic syndromes in humans

**DOI:** 10.1186/s12944-017-0489-3

**Published:** 2017-05-30

**Authors:** Muhmmad Asim Shabbir, Moazzam Rafiq Khan, Muhammad Saeed, Imran Pasha, Anees Ahmed Khalil, Naila Siraj

**Affiliations:** National Institute of Food Science and Technology, University of Agriculture, Faisalabad, 38000 Pakistan

**Keywords:** Punicic acid, Conjugated linoleic acid, Conjugated linolenic acid, Fatty acids, Antioxidant

## Abstract

Punicic acid, a bioactive compound of pomegranate seed oil has gained wide attention for their therapeutic potential. Different studies conducted on animal and human models have revealed that punicic acid is very effective against various chronic diseases. Substantial laboratory works has been carried out to elaborate punicic acid effectiveness and mechanism of action in animals. The intention of this review article is to explore the facts about the clinical trials of punicic acid and to discuss different future strategies that can be employed to use it in human clinical trials. Although punicic acid may represent a novel therapeutic unconventional approach for some disorders, still further experimental studies are required to demonstrate its effects in human beings.

## Background

Fruits and vegetables transformation into valuable products produces a huge amount of by-products that are considered to have a lot of bio-active compounds [1]. Pomegranate transformed to various edible products mainly fresh seeds, juice, nectars, jams and jellies. During the processing of pomegranate a large quantity of pomegranate waste is produced which has lots of nutritional components but mostly this waste is dumped off that creates environmental pollution and is also the wastage of nutritional components. In last few years, a huge excerpt of research work is conducted on the industrial by-products of pomegranate and found that these by-products have antioxidant, anti glycemic potential and antimicrobial activity. The multifarious reported functional attributes of pomegranate make it a unique fruit from other fruits. Due to the higher antioxidant potential of pomegranate fruit and its other parts e.g. juice, seed, peel etc., it has been reported that this fruit has advantageous health benefits for human body. This encouraged the nutritionists and scientists to find out more potential and beneficial bioactive ingredients for nutraceutical and food industry applications [2].

Pomegranate processing produces bagasse as a by-product after juice extraction that can be used as a valuable ingredients in other edible products but still its application is scarce. However, the research conducted in past revealed that the pomegranate seed residues (PSRs) have the potential to use in food products for different intentions. PSRs composed of sterols, polyphenols, tocopherols and octadecatrienoic acid (punicic acid). PSRs are the richest source of rare and unique pomegranate seed oil (PSO), an exotic, fruity aroma oil and used in aromatherapy with healing and the antioxidant potential for skin and breast cancer as concluded from different studies. PSO extracted by various extraction methods including organic solvent extraction, supercritical CO_2_ (SC-CO_2_) extraction and cold press extraction. Cold press extracted PSO has better physiochemical and nutritious quality as compared to other extraction methods [3, 4] with no impacts on environment. In SC-CO_2_ extraction method, the extraction pressure is the cardinal factor to reduce the PSO production [5]. PSO possessed the elevated level of punicic acid (PA) and tocopherol contents that decreased slightly with increase in temperature and pressure. However, an effective intelligent system was developed to investigate the effect of temperature and pressure during SC-CO_2_ on PSO yield and described that properly developed radial basis function and back-propagation neural network could be useful in predicting the effect of temperature and pressure on PSO yield in SC-CO_2_ extraction process [6]. PSO extracted through hexane by soxhlet method has superior quality as compared to subcritical propane and SC-CO_2_ extracted oil. Subcritical propane and SC-CO_2_ extraction method showed the efficiency up to 77% and 59% of total PSO as compared to extracted by soxhlet method [7].

PSO extracted through ultrasonic-assisted extraction method with optimized condition for best yield at solid-liquid ratio of 1:12 g/mL with extraction temperature 51°C at 70W for 40 minutes and 19% PSO yield with major fatty acids PA (65%), linoleic acid (10%) and oleic acid (9%) [8]. PSO showed the extraction efficiency in various solvents as followed: petroleum ether>n-hexane>ethyl acetate>ether>acetone>isopropanol [9]. Some other parameters such as ultrasonic power, time for extraction, solids: solvent ratio, and temperature were optimized with petroleum ether by surface response methodology. PSO extracted through ultrasonic assisted extraction showed superior quality and higher yield as compared to supercritical and soxhlet extraction methods. The effect of different extraction methods on the total phenolic contents extracted from pomegranate seeds of the Malas variety from Iran was studied and reported that different experimental conditions with supercritical extraction method can affect the phenolic contents. The temperature, pressure, and volume of modifier inversely proportional to phenolic extraction [10].

## Punicic acid (PA)

Various researchers have reported pomegranate as a functional fruit owing to presence of wide range of phytochemicals in it [11]. Purposely, antioxidative properties of juice, peel and seed have been examined for their therapeutic potential, which has prompted the nutritionists to further explore their nutraceutical and industrial application [2]. Its underutilized seed and peel portion, normally known as agro-waste obtained during industrial processing of pomegranate juice, are gaining attention of researchers now a days due to presence of array of nutraceutics in them. Abundant presence of pomegranate seed is of keen interest to scientist because of rich composition of oil [12]. Pomegranate seed oil possesses rich polyunsaturated fatty acid composition, predominantly comprising of punicic acid (~55%) as characterized by Melo [13]. This review summarizes PA and its role as a nutraceutical health substance.

PA is also recognize as “*Trichosanic acid”* with molecular formula C_18_H_30_O_2_ while its molar mass is 278.43 g/mol with melting point of 44-45^o^C. Punicic acid is an isomer of conjugated α-linolenic acid and a ω-5 polyunsaturated fatty acid which have structural resemblance with conjugated α-linolenic and linoleic acid, for instance number of double bonds and atomic arrangement. Due to health benefits associated with these fatty acids, scientists are showing great interest in exploring functional and nutraceutical properties of punicic acid against various metabolic ailments [14, 15].

The International Union of Pure and Applied Chemistry (IUPAC) designated its names as *9Z, 11E, 13Z-octadeca-9, 11, 13-trienoic acid* on the basis of its three double bonds (*cis*9, *trans*11 *and cis13*). Principally, it is an isomer of conjugated linoleic acid (c9t11) having a double bond on its tail side. Other isomers of lenolic acid are catalpic acid (*trans*9, *trans*11, *cis*13), alpha-eleostearic acid (*cis*9, *trans*11, *trans*13), calendic acid (*trans*8, *trans*10, *cis*12) and jacaric acid (*cis*8, *trans*10, *cis*12). PA is named after its principle source pomegranate (*Punica granatum* L.). Among all the sources of PA it is most abundantly present in pomegranate seed oil (PSO). The other sources for PA are snake gourd seed oil [16] and *Trichosanthes kirilowii* Maxim (TK) seeds (Table 1) containing 32 to 40% PA out of total seed weight [50: 9: 32]. Whereas, fatty acid profile of PSO contains conjugated linoleic acid up to 74–85 % PA [17] and remaining 14-25% are its isomers [18]. PA could be chemically synthesized by dehydration and isomerization of secondary oxidation products of linoleic and alpha-linolenic acids [19, 20]. Primarily, PA is reported to be effective against ailments like obesity, diabetics, inflammation, metabolic syndromes in various in vivo experiments (Table 2) that’s why pomegranate seed lipid portion (principal by-product) was studied after juice extraction as lipolytic enzymes deactivate during thermal processing [21].

**Table 1:** Sources of punicic acid

Sources of punicic acid (PA)		Authors
Pomegranate (Seed oil)		[74]
Saturated	10%	
Mono-unsaturated	10%	
Di-unsaturated	10%	
Punicic acid and isomer (C18: 3-9c,11t,13c)	70%	
*Trichosanthes kirilowii* (seed oil)		[53]
Saturated	7.50%	
Mono-unsaturated	22.91%	
Di-unsaturated	32.70%	
Punicic acid and isomer (C18: 3-*9c,11t,13c*)	35.89%

**Table 2:** Summary of health effects of punicic acid (PA) on animal and human model

Level of PA	Study model system	Mechanism of activity	Outcomes	Authors
5% punicic acid	ICR CD-1 mice	↑ CPTI activity	Decreased perirenal and epididymal fat	[46]
5% pomegranate seed (punicic acid)	OLEFT rats	↓ ∆9 desaturase activity	Adipose tissues weight reduction	[44]
1% punicic acid	C57Bl/J6 mice	-	Adipose tissues weight reduction	[17]
10-100 μM mixture of CLnA isomers (*cis*-9,*trans*-11,*cis*-15, and *cis*-9,*trans*-13,*cis*-15)	3T3-L1 cells	↑ HSL and ATGL gene expression	Decreased triglyceride content	[75]
10 and 50 μg/mL pomegranate seed (punicic acid)	3T3-L1 cells	↓ PPAR_Ƴ_ and C/EBP_β_↓ FAS	Decreased adipogenesis and preadipocyte differentiation	[56]
Punicic acid	C57Bl/J6 mice	-	Insulin sensitivity enhanced in peripheral area	[46]
Punicic acid	3T3-L1 cells and obese mice	PPARα and PPARβ activation	Improved glucose tolerance, with diabetes improvement	[76]
Punicic acid	HepG2 cells	↓ apoB100 secretion. Inhibition of stearoyl CoA desaturase	↓ Plasma triacylglycerides. Upgraded saturated/monounsaturated fatty acid ratio	[47]
Catalpic acid, jacaric acid, calendic acid, eleostearic acid and punicic acid,	Microsomes from sheep vesicular glands	Inhibition of cyclooxygenase activity (inhibition of prostaglandin synthesis)	Anti-inflammatory activity	[77]
Punicic acid	Sheep	Inhibit cyclooxygenase and lipoxygenase activity	Anti-inflammatory activity	[78]
Pomegranate extract (punicic acid)	3T3-L1 cells	PPARƳ receptor activator and agonist (inhibit NF-κβ expression, declined serum IL-6 and TNF-α)	Decreased chronic inflammation	[76]
Alpha-Eleostearic and punicic acid	Diabetic rats	Inhibit NF-κβ expression. Declined serum IL-6 and TNF-α.	Anti-inflammatory activity	[37]
Punicic acid (70% pomegranate seed oil)	-	Decreased expression PPARγ and C/EBPs, and fatty acid synthase	Suppresses adipocyte differentiation and lipid accumulation	[56]
Punicic acid	Ovariectomized (OVX) mice	Down-regulate the expression of osteoclast differentiation markers and RANK-RANKL downstream signaling targets in osteoclast-like cells (RAW264.7)	Improved bone mineral density and prevented trabecular micro-architecture impairment	[63]
Dietary mono-conjugated alpha-linolenic acid isomers	Neonatal pig	-	Safe for animals	[72]
Pomegranate seed oil	Rats	-	Improved insulin secretion	[48]

## Metabolism of punicic acid

**Fig. 1:**
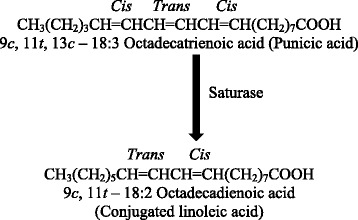
Metabolism of punicic acid

Numerous pharmacokinetics studies have been carried out on animals to assess the metabolism and bioavailability of PA. Results of efficacy trials revealed substantial evidence that PA is readily metabolized to circulating conjugated linoleic acid (c9t11) as depicted in Fig. 1 [22, 23]. Similarly, de Melo et al*.* [15] elucidated that oral administration of PA to rats for a time period of 24 hours conferred the metabolism of PA to 9c, 11t-conjugated linoleic acid (CLA) in plasma and other organs of the rats like kidney, liver, brain, heart and adipose tissues. 9c, 11t-CLA plays biologically significant role in maintaining the human body and is considered as an important natural resource of CLA via breakdown of CLnA. In a human trial conducted for 28 days, ingestion of *Trichosanthes kirilowii* (TK) seed kernels comprising 3g PA/day followed by 7 days feeding on sunflower seed kernels eventually increased the level of PA (c9t11c13) in membranes of plasma and red blood cells *i.e.* 0.47% and 0.37% correspondingly. Similarly, concentration of 9c, 11t-CLA elevated from 0.05 to 0.23% and 0.03 to 0.17% in plasma and red blood cell membranes, respectively.

It was investigated in different studies that uptake rate of CLnA vary in Caco-2 cells when distribution and conversion of CLnA (punicic, α- and β-eleostearic, and catalpic acid) to CLA is premeditated. Variation in the conversion efficiency is due to the difference in the structure of ∆13 double bond of CLnA. The CLnA distribute between neutral and phospholipids and this distribution depends upon the number of *trans* double bonds [24]. Nevertheless, another theory also exists about the conversion of CLnA to CLA. Accordingly, another theory explicates the metabolic conversion of CLnA to CLA is due to ∆13 double bond saturation reactions catalyzed by an enzyme *i.e.* nicotinamide adenine dinucleotide phosphate (NADP) that is known as a unique enzyme for conjugated trienoic acid reorganization [25].

In another study, an equi-molar mixture of conjugated linoleic acid (CLnA) (C18:3-9*c*, 11*t*, 15*c* and C18:3-9*c*, 13*t*, 15*c*) and rumenic acid (RA 18:2-9*c*, 11*t* ) was orally directed to Wister rats as free fatty acids (FFA) and triacylglycerol (TAG). Both isomers (RA and CLnA) were bio transformed completely in tissues in a similar fashion [26]. Mice supplemented with 1% PA for six weeks have higher concentration of ω-3 in liver phospholipids than the mice supplemented with 1% alpha-eleostearic acid [27]. Another experiment revealed similar results when diet was supplemented with 0.5% PA (PSO) [28]. When absorption and metabolic pathways of PA and alpha-eleostearic acid (α-ESA) was observed via a lipid absorption assay in rat intestine lymph, it was found that some amount of these isomers was converted to CLA rapidly and some remained intact in the intestines [29]. Comparatively, PA accumulates in higher amounts in tissues as compared to α-ESA and the conversion rate of α-ESA to 9*c*, 11*t*-CLA is also higher than PA in liver [27]. The maximum conversion rate of PA was 76% and 54.5% while highest conversion rate for α-ESA was 91.8% for adipose tissues, 91.4% for spleen, 90.7% for kidney and 84.6% for heart [27].

## Anti-oxidant potential of PA

Considering the side effects associated with usage of synthetic anti-oxidants and increase in oxidative stress disorders, use of anti-oxidants from natural sources are gaining importance [30]. Numerous studies revealed that PA is biologically active in hampering the hazards associated with oxidative mechanisms [14, 31]. To evaluate the anti-oxidative potential of PA against lipid peroxidation, various concentrations of PA (snake gourd seed oil) were supplemented to rats through diet after blending it with soybean. A momentous decrease was observed in body weight gain, energy consumption, triglyceride (TG), total cholesterol (TC) and TC/HDL ratio in high fat induced mouse model for five (05) weeks when administered by pomegranate leaf extract [32]. PA showed pro-oxidative (1.2%), anti-oxidative (0.6%) activity and significantly decreased the TC, low density lipoprotein cholesterol (LDL-C) when incorporated as compared to control diet [33]. PA and alpha-tocopherol (α-AT) were tested for its effects against lipid peroxidation in alloxan-induced diabetes mellitus rats. Significant lowering trend was observed in LDL and erythrocyte lipid peroxidation with highest reduction was noticed in liver & membrane peroxidation due to combined effect of 0.25% PA + 0.15% AT [34].

Activities of anti-oxidative enzymes in liver homogenates, brain, plasma and erythrocytes were altered by inducing sodium arsenite [35, 36]. Owing to supplementation of CLnA isomers, the activity of catalase (CAT), super-oxidase dismutase (SOD) and glutathione per-oxidase (GPx) were increased while decreasing trend was observed in nitric oxide (NO) synthase. However, α-ESA was found more effective in decreasing oxidative stress as compared to CLnA. When these isomers were studied against streptozotocin-induced diabetes in albino rats, same results were observed as in above mentioned studies against oxidative stress induced by sodium arsenite [37]. More distinct synergistic effect was observed when both of these isomers were applied in combination [38].

CLnA isomers improved the renal oxidative stress release and showed attired synergistic effects [4, 39]. The supplementation of mixture having both isomers of CLnA helped to improve the fatty acid profile of renal system. A decrease was observed in oxidation with the supplementation of TK seed (3 g PA). The increased level of 8-iso-PGF2α was due to non-enzymatic peroxidation of arachidonic acid [40, 41].

## Anti-diabetic properties

Up till now, experimental studies conducted to test the effect of PA on serum lipid, glucose metabolism and insulin resistance have provided controversial results. Non-significant results were recorded for both body and tissue weight when diet containing 1% α-ESA and/or PA was administered for six weeks [42]. Similar results were obtained when the mice were fed for three weeks on diet containing 0.12% and 1.2% PSO [43]. Feeding rats on 1% PSO and 9% safflower oil diet for the period of two weeks gives non-significant results for abdominal white adipose tissue weights [44]. In another study, a diet constituted with α-ESA and PA was given to hamsters for 6 weeks and reduction in the liver tissue TG levels was observed but no significant results were found for serum TC [45]. However in some other experimental studies, the results were in contrast with the above findings [34, 46, 47]. Some scientists found significant effect of PA on the reduction of TC, apoB-100 and liver tissue TG levels.

Rats that were fed on PSO showed higher levels of insulin in serum and glutathione peroxidase (GOP) activity with no significant difference in blood glucose level as compared to control group [48]. Peony seed oil rich in PA showed reduction in glycosylated total serum cholesterol (TC), triglyceride (TG), glycosylated hemoglobin (HbA1C) level while an increase was observed in weight gain, high density lipoprotein cholesterol (HDL-C), serum insulin level, liver glycogen level in mice [49]. In another study, PSO was fed to rats for 21 days that increased total triacylglycerol (TAG) and phospholipid level in serum while no significant increase was observed in TC level [50]. As the level of PSO increases in diet, the level of PA also increases in epididymal, serum, liver and peri-renal adipose. When mice were supplemented with dietary genetically modified rapeseed oil (GMRO) at 0.25% weight of the whole diet, it not only lowered the body lipid ratio but also enhanced the liver lipid metabolism as compared to the same quantity of PA taken directly from pomegranate. PSO was consistent in substantially reducing the liver TG levels but no major effects were observed for serum TG, TC, LDL-C and HDL-C levels [13].

When PSO was administered to dyslipidemic patients for four weeks, serum concentration of TNF-α decreased from 15 to 13.08pg/ml in the PSO group [51]. Similar scenario transpired when PSO given to high fat induced patients for four (04) weeks. No change occurred in serum TC, LDL-C, glucose concentrations and body composition variables [52]. It was found that supplementation of PSO for 1 month in hyper-lipidaemic subjects had promising effects on lipid profiles including TAG and TAG: HDL-C ratio. In contrast, fasting serum glucose and insulin levels and sensitivity showed no momentous variation due to the *Trichosanthes kirilowii* Maxim. Diet supplementation analyzed by homeostasis model assessment-insulin resistance (HOMA-IR) [53].

However when PA was given to rats with diet-induced obesity, an improvement was observed in plasma glucose and insulin levels and glucose-normalizing capacity over a glucose tolerance test [38]. An improvement in insulin sensitivity in CD-1 mice was observed with PSO consumption (av. 61 mg/day), signifying that the threat of emerging diabetes type-II might be lessened [54]. Additionally, it was noticed that PSO consumption improves glucose and insulin sensitivity and fat diet-induced obesity in mice. When PA (1% PSO) was supplemented for the period of three months, ingestion of PA resulted in lipid lowering sequenced by reduction in body weight which ultimately reduced total body mass [17]. PA also improved the peripheral insulin sensitivity without effecting liver insulin sensitivity. So, supplementation of PA through dietary sources is helpful with reference to insulin resistance and fat induced obesity in mice, independent of changes in food intake or energy expenditure.

Xanthigen (a bioactive compound derived from pomegranate) is the precursor of PA and is well known for its lipid lowering potential in animals and humans although its mechanism of action is not yet completely known. Xanthigen potently and dose-dependently suppressed accumulation of lipid droplets in adipocytes compared to its individual components; fucoxanthin and PSO [55]. Various mechanisms are involved in overwhelming the triacylglycerides accretion and adipocyte differentiation by Xanthigen and can be used as potential remedy for the treatment of diabetic patients. Mixture of PA, xanthigen and fucoxanthin acid (70% in PSO) was fed to mice to investigate the inhibitory effect on the differentiation of 3T3-L1 pre-adipocytes. A decreased gene expression was observed which was regulating the distinction process that raises TG buildup in 3T3-L1 pre-adipocytes treated with punicic acid [56].

## Effect of punicic acid on molecular events

The most common physiological disorders all around the world like rheumatoid arthritis, inflammatory bowel disease, metabolic syndrome and atherosclerosis that are specified by the occurrence of extremely stimulated inflammatory cells like macrophages, neutrophils, monocytes and overproduction of pro-inflammatory mediators and reactive oxygen species (ROS). These diseases can be effectively controlled by some natural dietary supplements. PA (PSO) is effective against ROS/MPO-mediated tissue damage and reduces the neutrophil-activation [57]. PA has persuasive anti-inflammatory effect and can prove as a natural therapeutic agent (as therapeutic substitute) against various inflammatory diseases [14].

Molecular evidence with reference to in vivo tryouts showed that the uptake of PA regulates colonic PPAR-δ expression, the keratinocyte growth factor, the orphan nuclear receptor RORγ expression, suppresses colonic and M1 macrophage-derived TNF-α. PA also increased the levels of IL-17 and IFN-γ in CD8+T cells in the mesenteric lymph nodes (i.e., mucosal inductive sites). PA modulates mucosal immune responses and improves gut inflammation through PPAR-γ and -δ-dependent mechanisms [28, 58]. Likewise, dietary PA lowers fasting plasma glucose concentrations and improves the glucose-normalizing ability eventually suppressing NF-κB activation and TNF-α expression. Ultimately it regulates PPAR α- and γ-responsive genes in skeletal muscle and adipose tissue [17]. Experiments have also demonstrated that PA can bind and robustly activate PPAR-γ and increase PPAR-γ-responsive gene expression, finally improving diabetic and inflammatory status. Insulin resistance by TNF-α is associated with mitochondrial dysfunction in 3T3-L1 adipocytes and is ameliorated by PA, a PPARγ agonist [59].

In an in vitro study, meant at assessing the efficiency and activeness of certain CLnA isomers found in PSO as selective estrogen receptor modulators (SERMs), PA also inhibited estrogen receptors (ER) α and β at 7.2 and 8.8 μM, respectively; α-ESA inhibited ER α and β at 6.5 and 7.8 μM, respectively [28, 60]. Thus, both CLnA isomers are effective as SERMs. These outcomes specify that PA, rich in PSO and an effective SERM, might be used as a possible breast cancer chemo-preventive agent.

Saha and Ghosh [40] revealed that the diet supplementation with CLnAs (α-ESA and PA at 0.5 % total lipids) showed significant reduction of inflammation in diabetic albino rats induced by streptozotocin. They further elaborated that the above mentioned four weeks diet supplementation led to reduce inflammation by reducing the expression of inflammatory cytokines, such as TNF-α and IL-6, in blood and the expression of hepatic NF-κB (p65), once higher as a result of diabetes induction. Then again, one study also stated that the 400 mg of pomegranate seed oil (rich in PA) administrated twice daily in dyslipidemic patients had no effect on serum TNF-α [28, 51]. These findings divulge that the CLnAs in PSO and some other foods may be a promising therapeutic substitute approach for cancer and inflammatory diseases. The further studies should be carried out to attest these effects in humans [37, 57].

Neurodegenerative disorders that occur due to the accretion of some special mis-folded proteins like in Alzheimer's diseases occupy almost same pathological features of oxidative damage and neuronal death. PA plays a vital role in controlling these diseases [61]. To examine the effect of PSO for oxidative damage reduction, PSO was encapsulated in a nano-droplet form and was given to TgMHu2ME199K mice (used as a model subject for genetic prion disease). PSO as a high source of rare polyunsaturated fatty acid (PA) is now being considered as a sturdiest biological antioxidant. When nano-PSO was administrated to already sick mice, it not only overdue the disease appearance but the disease aggravation was also delayed. Brain analysis of these mice showed that PSO lessened the fat oxidation and neuronal damage although it did not reduce PrPSc accretion. So, it is clear that PSO is a strong neuro-protective agent and is not only effective against the vulnerable subjects but also for those already experiencing neurodegenerative disorders. In the field of pharma, this kind of formulations can be helpful in ameliorating neurodegenerative disorders [62]. Certainly, nano-PSO is considered as a safe reagent and can be utilized as safe food supplement in different therapies.

Osteoporosis occurrence is increasing day by day and gaining importance as a great threat to long and healthy life expectancy. For the preclusion of osteoporosis, several strategies were developed. PSO is highly effective against inflammatory and oxidative processes as these processes are involved in osteoporosis. A study conducted on ovariectomized (OVX) mice model, supplemented with 5% PSO diet showed that PSO fed mice have significantly improved bone mineral density with prevention of trabecular micro-architecture impairment by involving osteo-clastogenesis inhibition and osteo-blastogenesis improvement. Thus PSO can be suggested as preventive measure against osteoporosis [63].

## Food Applications

Pomegranate seeds considered as the waste product after the processing of fruit into its various products. Pomegranate seed oil and peels were incorporated into ice-cream to enhance the functional properties and significant changes were observed in product pH, acidity and color while milk fat replacement with PSO improved the fatty acid profile by increasing the conjugated fatty acid contents with elevated antioxidant and anti-diabetic properties due to the phenolic contents [64]. Punicalagins and PA from peel and seed provide the health benefits with improvement in functional properties. Pomegranate seed ethanolic extract (PSEE) possessed the anti-proliferative and antioxidant effects against hormone-dependent prostate carcinoma and human breast cancer cell lines and considered as nutraceutical/functional food ingredient to prevent carcinogenic diseases [65].

Pomegranate seed extract useful in replacing artificial antioxidant in meat products to increase the oxidative stability of such products. Devatkal et al. [66] incorporated the citrus rind powder and pomegranate rind and seed extracts in meat patties. Highest antioxidant effect was observed in pomegranate seed extract. According to such studies, it is believed that industrial by-products of fruits and vegetables are potential source of antioxidant. Pomegranate seed extract helps to reduce the formation of heterocyclic aromatic amines in meat products cooked by various methods with different temperatures and heat [67] thus pomegranate seed useful in producing safe product.

Mohagheghi et al. [68] incorporated PSO (PSO-in-water emulsion) as functional ingredient in juice and beverages with varying concentration of gum Arabic and evaluated the effect on emulsion stability index, droplet size distribution, and turbidity loss rate. The consequences manifested that the beverage emulsions behaved as Newtonian fluids, and this study begins new glimpse to produce relatively stable PSO-in-water emulsions as functional ingredient in beverage industry. Encapsulation of PSO and its application in skimmed milk powder as encapsulating agent was studied by [69].

## Safety evaluation

PSO has been consumed for long enough due to its safety as cold pressed oil and is considered an effective measure to reduce the occurrence and incidence of tumor and multiplicity. It is also evident from different ex vivo and in vivo research trials using mice and rats as model subjects. However, there is still very little information is present about the safety and toxic concern of PSO other than the above mentioned evidences. PA is a non-toxic, natural, orally active food ingredient known to humans and consumed by humans for centuries [70, 71]. Possible mutagenicity of PSO was evaluated by means of in vitro and in vivo toxicity of PSO in Wistar rats [72]. During the presence or absence of metabolic activities, PSO can neither be clastogenic nor mutagenic in nature. No adverse effects of PSO were observed at a dose level of 4.3 g/kg body weight/day but at higher concentration (150,000 ppm) of PSO, hepatic enzyme activities are disturbed mainly due to increase in liver weight and liver-to-body ratio.

A short-term safety evaluation of dietary mono-conjugated alpha-linolenic acid isomers was executed using a pig model. The outcome of the experiment revealed that intake of mono-CLNA is safe in neonatal pigs for short-term time period. The safety of a mixture of two mono-CLnA c9-t11- c15-18:3 + c9-t13-c15-18:3 isomers was assessed in comparison to other fatty acids having either one conjugated double-bond, an n-3 or n-6 PUFA structures (CLnA vs. CLA; CLnA vs. W3; CLnA vs. W6) and results revealed that a dietary provision of 1% of these mono-CLnA isomers for 2-weeks can be considered safe for the current animal model [73].

It was observed that during the metabolic activation process, PSO did not show either clastogenicity or mutagenicity up to 33 μg/mL through chromosome aberration test or 5000 μg/plate through Ames test [74]. Rats fed on 2 g PSO/kg body weight did not showed non-significant toxicity effect. According to the OECD 423 test guidelines, the LD50 cut-off value supposed to be higher as compared to 5 g/kg body weight and PSO could be considered safe food without any labelling or classification requirement. In one 28 days oral toxicity study, PSO was administered to male and female rats at the rate of 150000 ppm with mean intake of 1.39 g PSO/kg body weight/day and it was observed that liver functioning enzymes improved in plasma followed by accretion of liver to body weight ratio. These results could be happened might be due to physiological actions of high dose of PA. According to the above mentioned data, PSO (PA) did not possess any toxic effect at such an elevated level as it’s not a part of normal diet at such high level. The no observable adverse effect level (NOAEL) of PA was reported at 50000 ppm as PSO, which is equivalent to 4.3 g PSO/kg body weight/day.

## Future concerns

The emergent data of clinical trials on animals and human models have explored that PA is as an effective bioactive compound that can be operative for human well-being for improved health eminence and chronic diseases preclusion. A minute single dose of PA is sufficient to integrate the physiological health effect within weeks or months with characteristic health changes particularly in clinical trials with compromised health prestige. The outward short-term effect of CLnA isomers supplementation in both liver and brain indicates that long-term toxicology trials in animals should be considered before moving to comprehensive and long-term studies. However, there is no evidence of long term supplementation effects on health related consequences, so it is justified that its effect must be investigated before further supplementation for long term time periods in animal models and before moving to clinical trials on humans. PA may possibly be used as a promising alternative in developing different new strategies for nutritional management and health complications. Further, to explore the potential of PA in improving human health, a continue stream line of research work is needed.

## Conclusion

Pomegranate seed oil (PSO) is abundantly comprised of punicic acid (PA), an isomer of conjugated linolenic acid (CLnA) as confirmed by recent studies around the world. Considering the beneficial aspects of PA in various studies, PSO recommended as a nutraceutical and/or functional ingredient for food products. However, various studies on the bioactive aspect of PA, there are still some ambiguous and/or antithetical studies also. In some studies, the output of PA showed different results in animal models as compared to the human model. There is demand for the PA studies in human models as these kinds of studies are scarce. In this way, the ultimate mechanism of PA on improving human health can be determined. Furthermore, the impact of the intake of PA (CLnA) as functional food on human body metabolism and other physiological health effects can be understood and finally the safety recommendations for the intake of PA can be developed.
